# Acrolein Impairs the Cholesterol Transport Functions of High Density Lipoproteins

**DOI:** 10.1371/journal.pone.0123138

**Published:** 2015-04-07

**Authors:** Alexandra C. Chadwick, Rebecca L. Holme, Yiliang Chen, Michael J. Thomas, Mary G. Sorci-Thomas, Roy L. Silverstein, Kirkwood A. Pritchard, Daisy Sahoo

**Affiliations:** 1 Department of Medicine, Medical College of Wisconsin, Milwaukee, Wisconsin, United States of America; 2 Department of Biochemistry, Medical College of Wisconsin, Milwaukee, Wisconsin, United States of America; 3 Blood Research Institute, Blood Center of Wisconsin, Milwaukee, Wisconsin, United States of America; 4 Department of Biochemistry, Wake Forest School of Medicine, Winston-Salem, North Carolina, United States of America; 5 Department of Pathology, Wake Forest School of Medicine, Winston-Salem, North Carolina, United States of America; 6 Department of Surgery, Children’s Research Institute, Milwaukee, Wisconsin, United States of America; 7 Cardiovascular Center, Medical College of Wisconsin, Milwaukee, Wisconsin, United States of America; Innsbruck Medical University, AUSTRIA

## Abstract

High density lipoproteins (HDL) are considered athero-protective, primarily due to their role in reverse cholesterol transport, where they transport cholesterol from peripheral tissues to the liver for excretion. The current study was designed to determine the impact of HDL modification by acrolein, a highly reactive aldehyde found in high abundance in cigarette smoke, on the cholesterol transport functions of HDL. HDL was chemically-modified with acrolein and immunoblot and mass spectrometry analyses confirmed apolipoprotein crosslinking, as well as acrolein adducts on apolipoproteins A-I and A-II. The ability of acrolein-modified HDL (acro-HDL) to serve as an acceptor of free cholesterol (FC) from COS-7 cells transiently expressing SR-BI was significantly decreased. Further, in contrast to native HDL, acro-HDL promotes higher neutral lipid accumulation in murine macrophages as judged by Oil Red O staining. The ability of acro-HDL to mediate efficient selective uptake of HDL-cholesteryl esters (CE) into SR-BI-expressing cells was reduced compared to native HDL. Together, the findings from our studies suggest that acrolein modification of HDL produces a dysfunctional particle that may ultimately promote atherogenesis by impairing functions that are critical in the reverse cholesterol transport pathway.

## Introduction

HDL is a multi-functional particle that participates in a variety of athero-protective roles that include promotion of endothelial homeostasis and inhibition of monocyte adhesion (reviewed in [1]). Arguably, HDL’s most important function is in preventing cholesterol accumulation in the vessel wall via the reverse cholesterol transport (RCT) pathway, where HDL is responsible for transporting cholesterol from peripheral tissues to the liver for excretion [2]. Delivery of cholesterol into the liver occurs by HDL binding to scavenger receptor class B type I (SR-BI), a highly glycosylated cell-surface receptor that mediates selective uptake of HDL-cholesteryl esters (CE) into the cell [3–5]. It has been suggested that HDL and SR-BI must be properly aligned in order to achieve efficient CE transfer [6], a process that requires the extracellular domain of SR-BI [7–10]. SR-BI is highly expressed in the liver and steroidogenic tissues [4,11], and is also present in macrophages where it has been suggested to play a role in free cholesterol (FC) efflux to HDL particles [12]. Altogether, the SR-BI/HDL interaction plays a crucial role in whole body cholesterol flux.

Oxidative stress plays a central role in the pathophysiology of atherosclerosis by inducing dyslipidemia, atheroma formation and endothelial dysfunction (reviewed in [13,14]). The role of oxidized low density lipoproteins (oxLDL) in promoting atherogenesis is well-established and has been studied for decades (reviewed in [15]). More recently, the oxidation of HDL by oxidative stress has been garnering much attention as we shift towards the concept that HDL function and cholesterol flux may be better predictors of cardiovascular risk than HDL-cholesterol levels [16,17]. Under oxidative stress, HDL is susceptible to modification by a large cohort of oxidants present *in vivo* (reviewed in [18]), such as metal ions [19,20], reactive aldehydes [19,21–23], and other products of endogenous oxidants [24,25], as well as environmental factors, such as poor diet and tobacco use [26,27]. These modifications to HDL may reduce or eliminate HDL’s athero-protective effects, leading to a “dysfunctional” particle (reviewed in [28,29]).

HDL proteins can be modified by the highly reactive α,β-unsaturated aldehyde, acrolein (CH_2_ = CH—CHO; 2-propenal) [23]. The human population is routinely exposed to acrolein as high levels of acrolein have been detected in cigarette smoke [30,31], as well as in many foods and beverages that include breads, cheese, donuts, coffee, beer, wine and rum [32]. Acrolein is also formed during the incomplete combustion of wood, plastics, gasoline and diesel fuel, as well as during the frying and re-heating of cooking oils ([33] and reviewed in [23]). Indeed, a recent study by DeJarnett *et al* shows a significant association between acrolein exposure and cardiovascular disease risk in humans [34]. Acrolein can also be produced endogenously as an end product of lipid peroxidation triggered by oxidative stress ([35,36] and reviewed in [37]) and as such, it is not surprising that acrolein has been detected in human atherosclerotic lesions [38,39]. Studies have shown that acrolein feeding can induce endothelial activation and atherosclerosis in apoE-null mice [40], as well as dyslipidemia where mice have elevated plasma cholesterol and triglyceride levels [41]. In other studies, acro-LDL was observed in plasma of patients with atherosclerosis and was shown to contribute to the development of atherosclerosis by promoting foam cell formation in THP-1 macrophages [42–44]. While acrolein appears to play a role in mediating processes that promote atherosclerosis, the mechanistic details of these pathways remain elusive.

Acrolein forms adducts with cysteine, histidine, and lysine residues [45], and its ability to modify apoA-I has been demonstrated [38,39]. Acrolein-modified apoA-I is also associated with impaired ATP-binding cassette transporter A1 (ABCA1)-mediated cholesterol efflux in BHK cells [39]. In this study, we move the field forward by determining the effects of acrolein modification of the entire HDL particle (acro-HDL) on cholesterol transport functions, and build on previous findings from acrolein modification of lipid-free apoA-I alone [38,39]. We have designed experiments to test our hypothesis that acro-HDL compromises HDL functions to generate a dysfunctional HDL particle that is unable to perform its athero-protective cholesterol-transport functions.

## Experimental Procedures

### Materials

The following antibodies were used: monoclonal anti-apoA-I (Santa Cruz Biotechnology, Inc), monoclonal anti-acrolein (Abcam), polyclonal anti-apoA-II (Abcam), polyclonal anti-SR-BI specific for the C-terminal or the extracellular domain (Novus Biologicals, Inc., Littleton, CO); peroxidase-conjugated bovine anti-goat secondary IgG (Santa Cruz Biotechnology, Inc.), peroxidase-conjugated goat anti-rabbit IgG (Amersham- GE Healthcare), anti-mouse (Amersham- GE Healthcare). [^3^H]Cholesteryl oleoyl ether (COE) was purchased from American Radiolabeled Chemicals, Inc (St. Louis, MO). [^125^I]Sodium iodide and [^3^H]cholesterol were purchased from Perkin-Elmer. Acrolein was purchased from Ultra Scientific (North Kingstown, RI). Aminoguanidine hydrochloride was purchased from Acros Organics (Morris Plains, NJ). All other reagents were of analytical grade.

### Plasmids

The human SR-BI coding region was cloned into the pcDNA3.1 vector (Invitrogen) to produce pcDNA3.1[hSR-BI] (herein referred to as SR-BI) [46].

### Cell Culture and Transfection

COS-7 cells were originally purchased from American Type Culture Collection (ATCC, Manassas, VA). Cells were maintained and transiently transfected using Fugene 6 as previously described [10]. Unless otherwise noted, cellular assays were performed 48 hours post-transfection or differentiation.

### Animals and primary cell culture

Wild-type C57BL6/J mice (12–18 weeks of age) were housed and bred in a pathogen-free barrier facility in accordance with federal and institutional guidelines. Mice were maintained on standard rodent chow under a normal light-dark cycle and housed and bred. All experimental procedures were approved by the Institutional Animal Care and Use Committee of the Medical College of Wisconsin. Mouse peritoneal macrophages were harvested as previously described [47] with slight modifications. Briefly, mice were injected intraperitoneally with 2 mL of 4% thioglycollate. Four days post-injection, mice were sacrificed by CO_2_ inhalation, followed by cervical dislocation, and injected with 10 mL warm PBS into the peritoneal cavity. The PBS/macrophage solution was collected and centrifuged at 1000 rpm for 8 minutes. Pelleted cells were resuspended in RPMI containing 10% fetal bovine serum, 2 mM _L_-glutamine, 50 units/mL penicillin, 50 μg/mL streptomycin, and 1 mM sodium pyruvate and plated at 1.5 x 10^6^ cells/well overnight at 37°C.

### Cell lysis

Transiently-transfected COS-7 cells were washed twice with cold PBS (pH 7.4) and lysed with 1% NP-40 cell lysis buffer containing protease inhibitors [10]. Protein concentrations were determined by the Lowry method as previously described [48].

### HDL labeling, cell association of [^125^I]HDL and uptake of [^3^H]HDL-COE

HDL was double-labeled with non-hydrolyzable [^3^H]COE and [^125^I]dilactitol tyramine as previously described [49]. Radiolabeled HDL preparations had [^3^H] average specific activity of 586.1 dpm/ng of protein and [^125^I] average specific activity of 313.6 dpm/ng of protein. COS-7 cells transiently transfected with empty pcDNA3.1 vector or wild-type SR-BI were assayed for cell association of [^125^I]-HDL and selective uptake of non-hydrolyzable [^3^H]-COE simultaneously, and selective uptake efficiency was calculated as described [9,50,51]. Competition assays were performed using similar methods, but with co-incubation of unlabeled lipoprotein and radiolabeled lipoprotein at the same time. Empty vector values were subtracted from wild-type SR-BI values and binding calculated. Statistical analyses were determined by t-test.

### Lipoproteins and their chemical modification

Human HDL and oxLDL were purchased from Biomedical Technologies, Inc. (Stoughton, MA). Native or radiolabeled HDL was incubated with 250 μM acrolein at 37°C for 24 h, unless otherwise noted. To control for potential oxidation of native HDL by overnight incubation at 37°C, unmodified HDL was also incubated at 37°C for 24 h, alongside acrolein-modified HDL for the same time period. Aminoguanidine hydrochloride (20-fold molar excess over acrolein) was added to the HDL solution (as well as unmodified HDL, as a control) to scavenge excess acrolein and experimental assays described were performed immediately.

### Measurement of free cholesterol efflux

COS-7 cells transiently expressing empty vector or wild-type human SR-BI were pre-labeled with [^3^H]cholesterol and assayed for FC release from cells to HDL or acro-HDL at 72-hours post-transfection as described [9,10]. Empty vector values were subtracted from wild-type SR-BI values. Following empty vector subtraction, all replicates were divided by the native HDL net % efflux for each experiment, thereby yielding native HDL normalization equal to 100% and acro-HDL as a percentage compared to native HDL. Statistical comparisons were calculated by one-way ANOVA with Bonferroni post-tests for all groups.

### Oil Red O staining of macrophages

Macrophages harvested from C57BL6/J mice were plated into 6-well plates containing coverslips (1 x 10^6^ cells/ well) in RPMI complete media. Media was replaced with fresh RPMI complete media 24 hours after plating. At 48 hours, macrophages were lipid-loaded by adding 50 μg/mL of oxLDL in 0.5% BSA/RPMI to each well and incubating overnight at 37°C. Cells were then washed with PBS and incubated overnight at 37°C with 50 μg/mL of either oxLDL, HDL, acrolein-modified HDL, or PBS in 0.5% BSA/RPMI media. Following lipoprotein treatment, macrophages were washed with cold PBS and fixed with 1% paraformaldehyde at room temperature for 30 minutes. Cells were washed and treated with 0.156% Oil Red O solution for 5 minutes at room temperature. Following washing, coverslips were mounted and imaged using bright-field microscopy (Nikon Eclipse TE2000-U). Percent foam cell formation was quantified by counting stained versus total cells in 7–8 fields.

### Macrophage lipid analysis by high-performance thin layer chromatography (TLC)

Macrophages were treated as described for Oil Red O staining. Following lipoprotein treatment, macrophages were washed with cold PBS and lipids were extracted with 2-propanol. Dried lipids were resuspended in chloroform: methanol (1:1 v/v) solution, spotted on glass TLC plates and separated using a hexane: isopropyl ether: acetic acid (65:35:2 v/v/v) solvent system. Bands were visualized using a cupric acetate/phosphoric acid solution (3% and 8% v/v, respectively) as described [52]. Intensities of bands corresponding to CE and FC were quantified using ImageJ software and percentages of each species were calculated.

### Mass spectrometry analyses of acrolein adducts

Intact HDL protein was analyzed with a Waters Q-TOF ApCI US quadrupole/time-of-flight mass spectrometer interfaced to a CapLC liquid chromatography apparatus through an Advion Nanomate electrospray source. A 25-μl aliquot of sample was diluted with 25 μl of methanol containing 2% formic acid). Five microliters were injected and proteins retained on a trapping column (3-μm- diameter PLRP, 300-Å pore diameter). After the salts were washed out for 9 min, the trapped protein was back flushed onto the reverse-phase analytical nanocolumn (0.1 mm by 50 mm packed with 3-μm-diameter PLRP having 100-Å pores) eluted at 450 nl/min. A 60-min gradient profile started with 98% solvent A (97:3, water:acetonitrile + 0.1% formic acid) for 10 min, a gradient to 80% solvent B (3:97, water:acetonitrile + 0.1% formic acid) at 25 min with a hold at 80% B for an additional 10 min. The electrospray spectrum was transformed using MaxEnt 1. Data were acquired from 600 to 1,900 m/z and were transformed with MassLynx software (version 4.0). Acquisition parameters were optimized for protein analysis, 2.4 s scan, 0.1 s interscan delay, cone voltage 55V, positive ion detection [53–55].

## Results

### Acrolein modifies HDL *in vitro*

**Fig 1:**
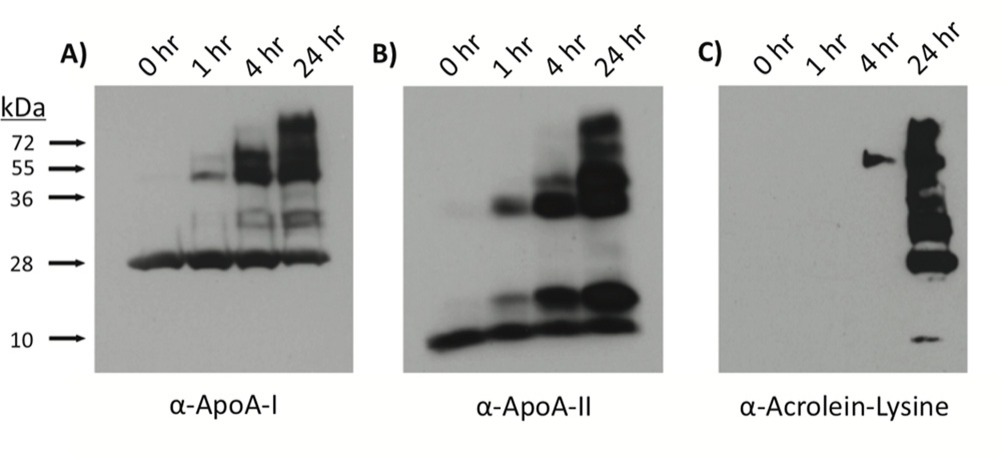
Immunoblot analysis verifies crosslinking and acrolein modification of HDL apolipoproteins. HDL was incubated with 250 μM acrolein for 1, 4, or 24 hours and proteins were separated on a 10% SDS-PAGE gel. Following transfer to a nitrocellulose membrane, proteins were visualized using antibodies directed against (A) apoA-I, (B) apoA-II or (C) α-acrolein-lysine. Data shown are representative of four independent modifications.

To test our hypothesis that acrolein modification of HDL impairs HDL function, we utilized immunoblot analyses to demonstrate that incubation of HDL with acrolein promoted crosslinking of the major apolipoproteins (Fig 1). Crosslinking of monomeric apoA-I (28 kDa; **Panel A**) or apoA-II (9 kDa; **Panel B**) was observed as early as after 1 hour of incubation with acrolein. Bands most likely represent apoA-I/apoA-I, apoA-II/apoA-II, as well as apoA-I/apoA-II crosslinks. **Panel C** demonstrates that acrolein adducts could be detected on HDL apolipoproteins as early as 4 hours after incubation. After 24 hours, acrolein adducts were observed on molecular weight species approximately 9, 28, 37, 56, 84, and 100+ kDa in size, corresponding to the predicted molecular weights of apoA-I and apoA-II monomers and multimers. ApoA-I/apoA-II crosslinked heterocomplexes are most likely represented by bands at ~ 38, 68 and 76 kDa, as has been reported by others [39].\

**Fig 2:**
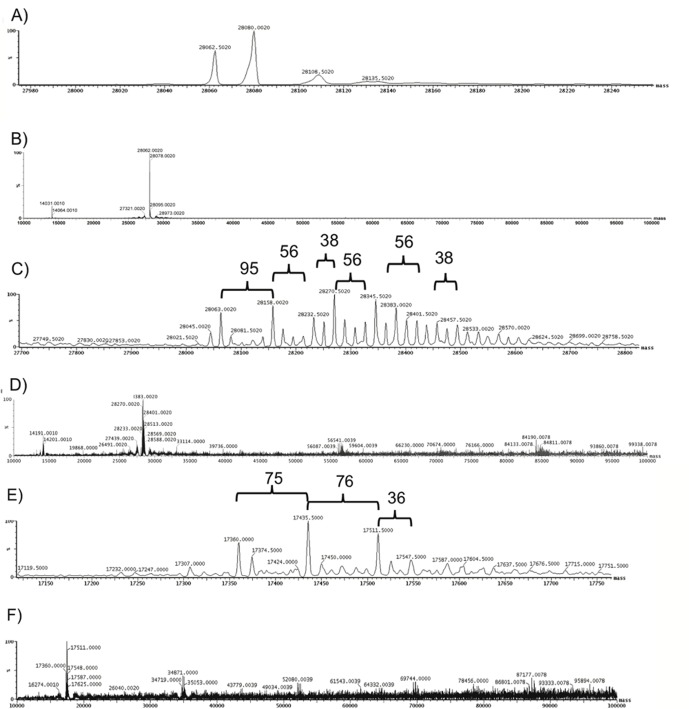
Mass spectrometry confirms the presence of acrolein adducts on HDL apolipoproteins. HDL was incubated with 250 μM acrolein for 24 hours. Intact protein was analyzed with a Waters Q-TOF ApCI US quadrupole/time-of-flight mass spectrometer interfaced to a CapLC liquid chromatograph through a Advion Nanomate electrospray source. Mass spectrometry analysis of (A, B) apoA-I on native HDL; (C, D) apoA-I on acro-HDL; and (E, F) apoA-II on acro-HDL are shown. Brackets indicate molecular weight shifts corresponding to acrolein adduct formation. An extended view of masses up to 100,000 Da are shown (Panels B, D, F).

We also used mass spectrometry to identify and characterize acrolein adduct formation on HDL particles (Fig 2). Higher molecular mass shifts of 38, 56, 76 and 95 Daltons likely represent adduct formation on lysine or possibly histidine residues, as previously shown [39]. These data also corroborate our immunoblot analysis of multimer species formation of apoA-I and apoA-II.

### Acrolein modification of HDL decreases efflux of free cholesterol from cells to HDL

**Fig 3:**
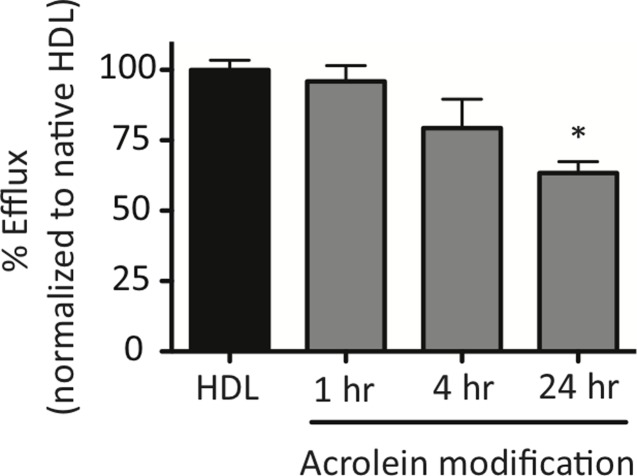
HDL modification by acrolein serves as a poor acceptor of free cholesterol effluxed from cells. COS-7 cells transiently expressing empty vector or human SR-BI were pre-labeled with [^3^H]cholesterol and cells were incubated for 4 hours with either 50 μg/mL native HDL or 50 μg/mL of HDL that was modified with acrolein for 1, 4, or 24 hours before the assay was performed. Radioactivity associated with the media and cells was quantified to calculate the efflux of FC out of the cells. Values represent the mean ± SEM of at least three independent experiments, each performed in quadruplicate. Data sets were normalized to native HDL (normalized value = 100%) following subtraction of empty vector values. *p < 0.001 as determined by one-way ANOVA.

Acrolein-modified HDL (acro-HDL) has been shown to decrease ABCA1-mediated efflux of FC to lipid-free-apoA-I in ABCA1-expressing BHK cells [39,56]. As SR-BI plays a role in stimulating FC efflux to mature HDL at the beginning of RCT [3], we hypothesized that acro-HDL’s ability to serve as an acceptor of FC released via SR-BI would be impaired. Compared to native HDL, longer periods of modification of HDL by acrolein generated a particle that was a less efficient acceptor of FC from SR-BI expressed in COS-7, with ~35% reduction in efflux capacity after 24 hours of acrolein modification (Fig 3).

**Fig 4:**
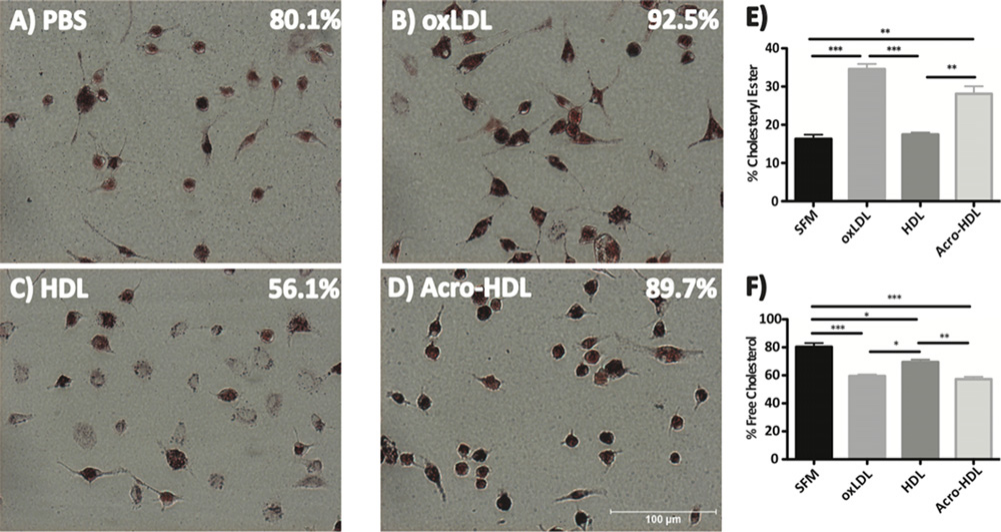
Acrolein-HDL is unable to stimulate efflux of free cholesterol from primary macrophages. Peritoneal macrophages harvested from C57BL6/J mice were pretreated with 50 μg/mL oxLDL for 24 hours at 37°C. Macrophages were then incubated with PBS or 50 μg/mL oxLDL, HDL or acrolein-modified HDL for an additional 24 hours at 37°C. Neutral lipids were visualized by Oil Red O staining (Panels A-D). Data are representative of 3 independent experiments. Percent foam cell formation was quantified by counting stained versus total cells in 7–8 fields. Cholesteryl ester (Panel E) and free cholesterol (Panel F) content were quantified using TLC. Values represent the mean ± SEM of three treatment replicates. Statistical analyses were determined by one-way ANOVA. *p<0.05, **p<0.01 and ***p<0.001

We also examined the ability of acro-HDL to serve as an acceptor of FC released from murine macrophages. Cholesterol-loaded mouse peritoneal macrophages were incubated with PBS or 50 μg/mL of oxLDL, native HDL or acro-HDL for 24 hours and percent foam cells were quantified (Fig 4). As expected, intense Oil Red O staining of macrophages was observed in the presence of oxLDL [57] consistent with further cellular accumulation of neutral lipids (**Panel B**). However, significantly less neutral lipid staining was observed after incubation with native HDL, consistent with efficient efflux of FC (**Panel C vs. Panel A**). Oil Red O staining was not decreased after incubation with acro-HDL, consistent with impaired release of FC; in fact, the macrophages displayed enhanced staining similar to cells incubated with oxLDL (**Panel D**). Furthermore, TLC analysis of lipids verified that acro-HDL treated macrophages had higher CE content (**Panel E**) and lower FC content (**Panel F**) compared to native HDL. These values were similar to those observed for oxLDL.

### Acrolein modification of HDL decreases SR-BI-mediated selective uptake efficiency of CE

**Fig 5:**
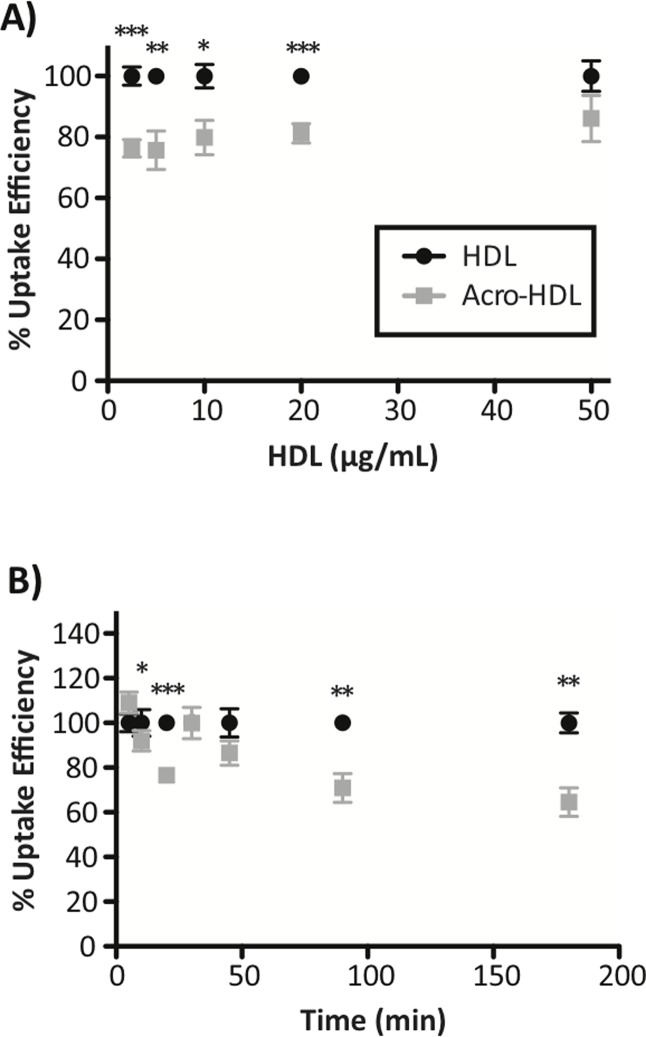
HDL modification by acrolein leads to decreased selective uptake efficiency of HDL-COE. SR-BI-expressing COS-7 cells were incubated with double-radiolabeled HDL or acro-HDL and selective uptake efficiency for each ligand was calculated as a function of ligand concentration (panel A; for 1.5 h at 37°C) or incubation time (panel B; ligand concentration of 10 μg/mL at 37°C). Values for binding to SR-BI and selective uptake of COE are shown in Table 1. Values represent the mean ± SEM of at least four independent experiments, each performed in triplicate. Data sets are presented following subtraction of empty vector values. Statistical analyses were determined by paired t-test. *p<0.05, **p<0.01 and ***p<0.001

Having shown that acro-HDL was an inefficient acceptor of FC from SR-BI-transfected cells and macrophages, we next hypothesized that acrolein modification would also impair the other key SR-BI-dependent function—selective uptake of HDL-CE, which is a critical step important in the hepatic phase of RCT. Using dual-radiolabeled HDL particles, we found, surprisingly, that more acro-HDL remained bound to SR-BI in transfected COS-7 cells than native HDL (Table 1). This trend was observed over different concentrations of acro-HDL, as well as with increasing amounts of time that acro-HDL was incubated on cells, as compared to native HDL. Native HDL had an apparent *K*_*d*_ of 15.8 μg/mL with a B_max_ of 220.2 ng HDL protein/mg cell protein, while acro-HDL had a higher *K*_*d*_ of 20.0 μg/mL with a B_max_ of 284.0 ng HDL protein/mg cell protein. Although acro-HDL displayed increased binding to SR-BI compared to native HDL, a corresponding increase in HDL-CE uptake was not observed (Table 1). Therefore, as compared to native HDL, selective uptake efficiency (i.e. the amount of HDL-CE selective uptake with respect to the amount of HDL binding) for acro-HDL was significantly reduced as a function of ligand concentration (Fig 5A) or incubation time (Fig 5B).

**Table 1:** Binding of native HDL and acro-HDL to SR-BI and selective uptake of COE.

		Binding to SR-BI(ng HDL protein/mg cell protein) ± SEM		Uptake of HDL-COE(ng HDL-COE protein/mg cell protein) ± SEM	
		HDL	Acro-HDL	HDL	Acro-HDL
**Lipoproteinconcentration(μg/mL)**	2.5	27.5 ± 3.6	29.3 ± 2.6	179.2 ± 19.8	147.8 ± 21.5
	5	54.1 ± 7.0	54.7 ± 4.1	324.0 ± 38.0	260.5 ± 35.4
	10	88.7 ± 9.2	108.9 ± 9.7 ***	551.6 ± 64.1	591.3 ± 61.6
	25	117.3 ± 7.6	125.7 ± 7.6	1149.6 ± 133.1	963.5 ± 105.8 *
	50	168.9 ± 5.8	205.4 ± 13.7 **	956.5 ± 153.6	1085.2 ± 164.9
**Time (min)**	5	18.0 ± 1.2	15.9 ± 1.7	5.3 ± 0.4	5.0 ± 0.4
	10	27.3 ± 1.8	24.2 ± 1.8	9.1 ± 0.7	7.5 ± 0.7 *
	20	35.1 ± 2.2	42.3 ± 0.9 **	16.0 ± 1.3	15.6 ± 1.5
	30	37.4 ± 2.4	39.4 ± 3.5	17.7 ± 2.7	18.0 ± 2.8
	45	44.3 ± 4.3	53.5 ± 2.3 *	30.3 ± 5.0	21.4 ± 2.2
	90	52.0 ± 4.1	59.7 ± 3.9 **	46.3 ± 7.8	37.1 ± 6.2 *
	180	45.8 ± 7.7	64.7 ± 3.1 *	74.3 ± 15.3	68.1 ± 10.6

COS-7 cells transiently transfected with empty vector (pcDNA3) or human SR-BI were incubated with native- or acrolein-modified [^125^I]/[^3^H]-COE-labeled HDL. Binding of radiolabeled HDL/acro-HDL to SR-BI and selective uptake of HDL-COE were measured as a function of ligand concentration (for 1.5 hours at 37°C) or incubation time (ligand concentration of 10 μg/mL at 37°C). Values represent the mean ± SEM of at least four independent experiments, each performed in triplicate. Data sets are presented following subtraction of empty vector values. Statistical analyses were determined by paired t-test.

*p<0.05,

**p<0.01

***p<0.001

**Fig 6:**
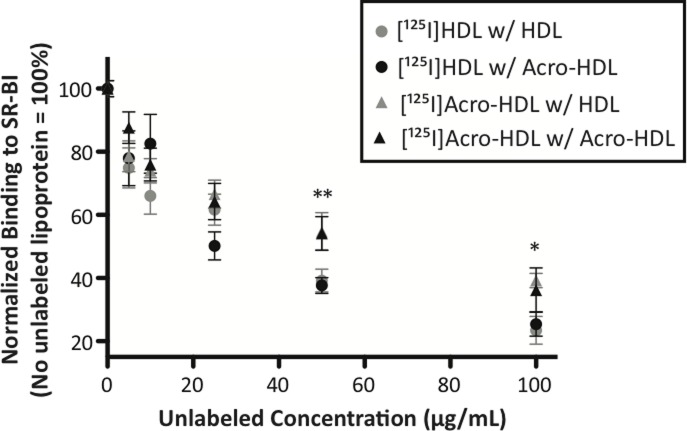
Competition of native HDL and acrolein-HDL for binding to SR-BI. COS-7 cells transiently transfected with empty vector (pcDNA3) or SR-BI were incubated with [^125^I]-labeled native HDL (circles) or acrolein-modified HDL (triangles) (10 μg/mL) in the presence of increasing concentrations of unlabeled native- or acrolein-modified HDL for 1.5 h at 37°C. Values represent the mean ± SEM of three independent experiments, each performed in triplicate. Binding of radiolabeled ligand was quantified and expressed as a percent of control in the absence of competitor, following subtraction of empty vector values. *p<0.05, **p<0.01

Since SR-BI bound more acro-HDL as compared to native HDL, we hypothesized that acro-HDL may compete with native HDL for similar binding sites on SR-BI. To test this hypothesis, a fixed concentration of either [^125^I]HDL or [^125^I]acro-HDL was added to SR-BI-expressing cells in the presence of unlabeled competitor. As shown in Fig 6, both HDL and acro-HDL were able to compete with radiolabeled HDL with similar apparent affinities. Both ligands were also able to similarly compete with radiolabeled acro-HDL. These data suggest that both HDL and acro-HDL share similar binding sites on SR-BI. Interestingly, we observed that both unlabeled ligands were less effective at competing off [^125^I]acro-HDL than [^125^I]HDL (compare triangles to circles), suggesting that it is more difficult to dissociate acro-HDL from SR-BI as compared to native HDL.

## Discussion

There has been a growing emphasis on investigating the role of dysfunctional HDL in atherosclerosis, with evidence suggesting that modifications to HDL proteins may play a role in the pathogenesis of cardiovascular disease [18,28,34,58]. Acrolein has been shown to play a role in promoting atherogenesis [40,41], but its mechanism of action has been poorly studied. Our data revealed that acrolein modification of HDL: (i) impairs the ability of HDL to serve as an acceptor of FC from cells and (ii) reduces the efficiency of SR-BI-mediated HDL-CE selective uptake. Together, these data suggest that modification of HDL by acrolein alters the ability of HDL to fully participate in reverse cholesterol transport. To our knowledge, the current report is one of the only to investigate how acrolein impairs HDL function as it pertains to processes related to reverse cholesterol transport, and significantly builds on available literature that only reports the effects of acrolein modification on lipid-free apoA-I function.

We verified that acrolein forms adducts with apoA-I and apoA-II and leads to protein crosslinking around the HDL particle. Formation of acrolein adducts on apoA-I has been characterized [22,39] and corroborate the patterns of migration we observe on our immunoblots, as well as the adduct formation and protein crosslinking detected by our mass spectrometry analyses. Acrolein modification of apoA-II has been observed by gel electrophoresis [22,39], yet to our knowledge, this is the first report of acrolein adducts on apoA-II detected by mass spectrometry. Shao *et al*. elegantly demonstrated that lysine residues of apoA-I are primarily targeted for modification by acrolein [39]. In the case of apoA-II, this apolipoprotein does not contain histidine residues, and it is unlikely that acrolein modifies the single cysteine that is known to participate in apoA-II homodimerization via disulfide bond formation [59]. Indeed, the monoclonal antibody used in our experiments was directed against acrolein-lysine adducts [39] and bands were clearly visible (Fig 1, Panel C). While bands most likely represent multimers of apoA-I, apoA-II or apoA-I/apoA-II, we cannot exclude the possibility that these apolipoproteins may also form crosslinks with other proteins that belong to the HDL proteome.

Acrolein-modified HDL, in contrast to native HDL: (i) serves as poor acceptor of FC from cells via SR-BI; and (ii) promotes higher neutral lipid accumulation in mouse macrophages as judged by Oil Red O staining. Efflux of FC from SR-BI-expressing COS-7 cells decreased after longer exposure of HDL to acrolein. Shao *et al*. reported that Lys-226 of apoA-I was the major site of acrolein modification, and was particularly important for ABCA1-mediated efflux [39]. Whether Lys-226 is also important for SR-BI-mediated efflux needs to be further explored. We also cannot exclude the possibility that in combination with decreased cholesterol efflux, incubation of macrophages with acro-HDL may facilitate cholesterol or even whole particle uptake into cells, similar to the behavior of oxLDL. Indeed, acro-HDL-treated macrophages had a significantly higher CE content than native HDL, closely resembling the pattern observed for oxLDL. As such, we are currently investigating the possibility that acro-HDL may promote CE delivery into macrophages.

Our data indicate that although more acro-HDL binds to SR-BI than native HDL, there was a statistically significant decrease in the selective uptake efficiency of HDL-CE. Our calculated *K*_*d*_ of 15.8 μg/mL and *K*_*m*_ of 13.6 μg/mL fall directly within the range previously reported for native HDL [9,60–62]. Our data indicate that acro-HDL has a higher B_max_ compared to native HDL (284.0 vs 220.2 ng HDL protein/ mg cell protein, respectively), suggesting an increase in the number of binding sites on SR-BI and/or increased affinity of SR-BI for acro-HDL. Increased SR-BI binding may result from the oxidation of HDL’s proteins and lipids, as has been observed for oxLDL which binds SR-BI with a higher affinity than native HDL [63]. As oxidized lipid composition plays a crucial role in oxidized lipoprotein binding to SR-BI [63], the possible modification of HDL lipids by acrolein warrants further investigation. Our data reinforce the concept that binding of HDL to SR-BI must be “productive” [6], where the receptor and ligand are precisely aligned in such a manner that supports the conformational changes that may be required for efficient selective uptake of HDL lipids. It is possible that acrolein-induced protein crosslinking and/or the presence of acrolein adducts around HDL may interfere with productive complex formation, thus causing a percentage of acro-HDL binding to be unproductive at different binding sites, or even promote tighter binding interactions. Indeed, our competition assays support the notion that acro-HDL has a stronger association with SR-BI than native HDL, suggesting that acrolein modification may prevent the HDL particles from dissociating from SR-BI and re-entering the circulation to participate in additional rounds of lipid transfer. This concept of impaired dissociation is also supported by our time course assays (Table 1 and Fig 5B), where despite increased binding of acro-HDL to SR-BI, the overall efficiency of HDL-CE delivery to cells is reduced and statistically significant. Whether similar observations can be made in a hepatocyte model that endogenously expresses SR-BI is currently being investigated.

Acrolein has been shown to impact lipoprotein function in other ways. For example, acrolein-modified HDL inactivated paraoxonase-1 activity [64], while addition of acrolein to human plasma was shown to reduce LCAT activity [22]. Further, acrolein modification of apoE impaired its ability to serve as a ligand for the LDL receptor [65]. It is important to note that we modified HDL with 250 μM, a concentration that is within the range and/or lower than what others have used for modification of apoA-I [22,39,64]. Even more importantly, we believe this concentration is within the realm of physiological conditions in habitual smokers, since acrolein concentrations in healthy non-smokers was found to be in the low micromolar range ([66], reviewed in [36]).

This low level of modification was able to promote adduct formation/apolipoprotein crosslinks, as well as changes in function of the HDL particle, suggesting that exposure to very low levels of acrolein is sufficient to impair cholesterol transport. While our studies focused on the effects of acrolein, our findings highlight the need to improve our understanding of how oxidative stress can impact HDL function. Further, identifying the molecular mechanisms by which dysfunctional HDL becomes pro-atherogenic is critical as we continue to develop therapies that prevent atherosclerosis and CVD.

## References

[1] Navab M, Reddy ST, Van Lenten BJ, Fogelman AM. HDL and cardiovascular disease: atherogenic and atheroprotective mechanisms. Nat Rev Cardiol. 2011; 8: 222–232. 10.1038/nrcardio.2010.222 21304474

[2] Glomset JA. The plasma lecithins:cholesterol acyltransferase reaction. J Lipid Res. 1968; 9: 155–167. 4868699

[3] Acton S, Rigotti A, Landschulz KT, Xu S, Hobbs HH, Krieger M. Identification of scavenger receptor SR-BI as a high density lipoprotein receptor. Science. 1996; 271: 518–520. 8560269 10.1126/science.271.5248.518

[4] Glass C, Pittman RC, Civen M, Steinberg D. Uptake of high-density lipoprotein-associated apoprotein A-I and cholesterol esters by 16 tissues of the rat in vivo and by adrenal cells and hepatocytes in vitro. J Biol Chem. 1985; 260: 744–750. 3918032

[5] Pittman RC, Knecht TP, Rosenbaum MS, Taylor CA Jr. A nonendocytotic mechanism for the selective uptake of high density lipoprotein-associated cholesterol esters. J Biol Chem. 1987; 262: 2443–2450. 2434485

[6] Liu T, Krieger M, Kan HY, Zannis VI. The effects of mutations in helices 4 and 6 of ApoA-I on scavenger receptor class B type I (SR-BI)-mediated cholesterol efflux suggest that formation of a productive complex between reconstituted high density lipoprotein and SR-BI is required for efficient lipid transport. J Biol Chem. 2002; 277: 21576–21584. 11882653 10.1074/jbc.M112103200

[7] Gu X, Trigatti B, Xu S, Acton S, Babitt J, Krieger M. The efficient cellular uptake of high density lipoprotein lipids via scavenger receptor class B type I requires not only receptor-mediated surface binding but also receptor-specific lipid transfer mediated by its extracellular domain. J Biol Chem. 1998; 273: 26338–26348. 9756864 10.1074/jbc.273.41.26338

[8] Connelly MA, de la Llera-Moya M, Monzo P, Yancey PG, Drazul D, Stoudt G, et al Analysis of chimeric receptors shows that multiple distinct functional activities of scavenger receptor, class B, type I (SR-BI), are localized to the extracellular receptor domain. Biochemistry. 2001; 40: 5249–5259. 11318648 10.1021/bi002825r

[9] Connelly MA, De La Llera-Moya M, Peng Y, Drazul-Schrader D, Rothblat GH, Williams DL. Separation of lipid transport functions by mutations in the extracellular domain of scavenger receptor class B, type I. J Biol Chem. 2003; 278: 25773–25782. 12730208 10.1074/jbc.M302820200

[10] Papale GA, Nicholson K, Hanson PJ, Pavlovic M, Drover VA, Sahoo D. Extracellular hydrophobic regions in scavenger receptor BI play a key role in mediating HDL-cholesterol transport. Arch Biochem Biophys. 2010; 496: 132–139. 10.1016/j.abb.2010.02.011 20219439 PMC2853188

[11] Pittman RC, Steinberg D. Sites and mechanisms of uptake and degradation of high density and low density lipoproteins. J Lipid Res. 1984; 25: 1577–1585. 6397563

[12] Ji Y, Jian B, Wang N, Sun Y, Moya ML, Phillips MC, et al Scavenger receptor BI promotes high density lipoprotein-mediated cellular cholesterol efflux. J Biol Chem. 1997; 272: 20982–20985. 9261096 10.1074/jbc.272.34.20982

[13] Pashkow FJ. Oxidative Stress and Inflammation in Heart Disease: Do Antioxidants Have a Role in Treatment and/or Prevention? Int J Inflam. 2011; 2011: 514623 10.4061/2011/514623 21860805 PMC3157078

[14] Bonomini F, Tengattini S, Fabiano A, Bianchi R, Rezzani R. Atherosclerosis and oxidative stress. Histol Histopathol. 2008; 23: 381–390. 18072094 10.14670/HH-23.381

[15] Steinberg D, Witztum JL. Oxidized low-density lipoprotein and atherosclerosis. Arterioscler Thromb Vasc Biol. 2010; 30: 2311–2316. 10.1161/ATVBAHA.108.179697 21084697

[16] Toth PP, Barter PJ, Rosenson RS, Boden WE, Chapman MJ, Cuchel M, et al High-density lipoproteins: a consensus statement from the National Lipid Association. J Clin Lipidol. 2013; 7: 484–525. 10.1016/j.jacl.2013.08.001 24079290

[17] Larach DB, deGoma EM, Rader DJ. Targeting high density lipoproteins in the prevention of cardiovascular disease? Curr Cardiol Rep. 2012; 14: 684–691. 10.1007/s11886-012-0317-3 22991041 PMC3517174

[18] Ferretti G, Bacchetti T, Negre-Salvayre A, Salvayre R, Dousset N, Curatola G. Structural modifications of HDL and functional consequences. Atherosclerosis. 2006; 184: 1–7. 16157342 10.1016/j.atherosclerosis.2005.08.008

[19] Salmon S, Maziere C, Auclair M, Theron L, Santus R, Maziere JC. Malondialdehyde modification and copper-induced autooxidation of high-density lipoprotein decrease cholesterol efflux from human cultured fibroblasts. Biochim Biophys Acta. 1992; 1125: 230–235. 1571368 10.1016/0005-2760(92)90050-6

[20] Raveh O, Pinchuk I, Schnitzer E, Fainaru M, Schaffer Z, Lichtenberg D. Kinetic analysis of copper-induced peroxidation of HDL, autoaccelerated and tocopherol-mediated peroxidation. Free Radic Biol Med. 2000; 29: 131–146. 10980402 10.1016/s0891-5849(00)00332-4

[21] Maziere JC, Myara I, Salmon S, Auclair M, Haigle J, Santus R, et al Copper- and malondialdehyde-induced modification of high density lipoprotein and parallel loss of lecithin cholesterol acyltransferase activation. Atherosclerosis. 1993; 104: 213–219. 8141845 10.1016/0021-9150(93)90192-w

[22] McCall MR, Tang JY, Bielicki JK, Forte TM. Inhibition of lecithin-cholesterol acyltransferase and modification of HDL apolipoproteins by aldehydes. Arterioscler Thromb Vasc Biol. 1995; 15: 1599–1606. 7583533 10.1161/01.atv.15.10.1599

[23] Bhatnagar A. Environmental Cardiology: Studying Mechanistic Links Between Pollution and Heart Disease. Circ Res. 2006; 99: 692–705. 17008598 10.1161/01.RES.0000243586.99701.cf

[24] Bonnefont-Rousselot D, Motta C, Khalil AO, Sola R, La Ville AE, Delattre J, et al Physicochemical changes in human high-density lipoproteins (HDL) oxidized by gamma radiolysis-generated oxyradicals. Effect on their cholesterol effluxing capacity. Biochim Biophys Acta. 1995; 1255: 23–30. 7893734 10.1016/0005-2760(94)00211-g

[25] Shao B, Heinecke JW. Impact of HDL oxidation by the myeloperoxidase system on sterol efflux by the ABCA1 pathway. J Proteomics. 2011; 74 (11): 2289–2299. 10.1016/j.jprot.2011.04.001 21501700 PMC3156866

[26] McCall MR, van den Berg JJ, Kuypers FA, Tribble DL, Krauss RM, Knoff LJ, et al Modification of LCAT activity and HDL structure. New links between cigarette smoke and coronary heart disease risk. Arterioscler Thromb. 1994; 14: 248–253. 8305416 10.1161/01.atv.14.2.248

[27] Aseervatham GS, Sivasudha T, Jeyadevi R, Arul Ananth D. Environmental factors and unhealthy lifestyle influence oxidative stress in humans—an overview. Environ Sci Pollut Res Int. 2013; 20: 4356–4369. 10.1007/s11356-013-1748-0 23636598

[28] Navab M, Reddy ST, Van Lenten BJ, Anantharamaiah GM, Fogelman AM. The role of dysfunctional HDL in atherosclerosis. J Lipid Res. 2009; 50 Suppl: S145–S149. 10.1194/jlr.R800036-JLR200 18955731 PMC2674720

[29] Otocka-Kmiecik A, Mikhailidis DP, Nicholls SJ, Davidson M, Rysz J, Banach M. Dysfunctional HDL: a novel important diagnostic and therapeutic target in cardiovascular disease? Prog Lipid Res. 2012; 51: 314–324. 10.1016/j.plipres.2012.03.003 22609245

[30] Johnstone RAW, Plimmer JR. The Chemical Constituents of Tobacco and Tobacco Smoke. Chem Rev. 1959; 59: 885–936.

[31] Witz G. Biological interactions of alpha,beta-unsaturated aldehydes. Free Radic Biol Med. 1989; 7: 333–349. 2673948 10.1016/0891-5849(89)90137-8

[32] Feron VJ, Til HP, de Vrijer F, Woutersen RA, Cassee FR, van Bladeren PJ. Aldehydes: occurrence, carcinogenic potential, mechanism of action and risk assessment. Mutat Res. 1991; 259: 363–385. 2017217 10.1016/0165-1218(91)90128-9

[33] Fullana A, Carbonell-Barrachina AA, Sidhu S. Comparison of volatile aldehydes present in the cooking fumes of extra virgin olive, olive, and canola oils. J Agric Food Chem. 2004; 52: 5207–5214. 15291498 10.1021/jf035241f

[34] DeJarnett N, Conklin DJ, Riggs DW, Myers JA, O'Toole TE, Hamzeh I, et al Acrolein Exposure Is Associated With Increased Cardiovascular Disease Risk. J Am Heart Assoc. 2014; 3. 10.1161/JAHA.114.000934 PMC4310380 25099132

[35] Izard C, Libermann C. Acrolein. Mutat Res. 1978; 47: 115–138. 415230 10.1016/0165-1110(78)90016-7

[36] Stevens JF, Maier CS. Acrolein: sources, metabolism, and biomolecular interactions relevant to human health and disease. Mol Nutr Food Res. 2008; 52: 7–25. 10.1002/mnfr.200700412 18203133 PMC2423340

[37] Jairam VU, K.; Narayanaswami V. Pathophysiology of Lipoprotein Oxidation, Lipoproteins- Role in Health and Diseases. Biochem Gen and Mol Biol. 2012: 383–408.

[38] Shao B, O'Brien K D, McDonald TO, Fu X, Oram JF, Uchida K, et al Acrolein modifies apolipoprotein A-I in the human artery wall. Ann N Y Acad Sci. 2005; 1043: 396–403. 16037261 10.1196/annals.1333.046

[39] Shao B, Fu X, McDonald TO, Green PS, Uchida K, O'Brien KD, et al Acrolein impairs ATP binding cassette transporter A1-dependent cholesterol export from cells through site-specific modification of apolipoprotein A-I. J Biol Chem. 2005; 280: 36386–36396. 16126721 10.1074/jbc.M508169200

[40] Srivastava S, Sithu SD, Vladykovskaya E, Haberzettl P, Hoetker DJ, Siddiqui MA, et al Oral exposure to acrolein exacerbates atherosclerosis in apoE-null mice. Atherosclerosis. 2011; 215: 301–308. 10.1016/j.atherosclerosis.2011.01.001 21371710 PMC3070047

[41] Conklin DJ, Barski OA, Lesgards JF, Juvan P, Rezen T, Rozman D, et al Acrolein consumption induces systemic dyslipidemia and lipoprotein modification. Toxicol Appl Pharmacol. 2010; 243: 1–12. 10.1016/j.taap.2009.12.010 20034506 PMC2922677

[42] Watanabe K, Nakazato Y, Saiki R, Igarashi K, Kitada M, Ishii I. Acrolein-conjugated low-density lipoprotein induces macrophage foam cell formation. Atherosclerosis. 2013; 227: 51–57. 10.1016/j.atherosclerosis.2012.12.020 23305793

[43] Obama T, Kato R, Masuda Y, Takahashi K, Aiuchi T, Itabe H. Analysis of modified apolipoprotein B-100 structures formed in oxidized low-density lipoprotein using LC-MS/MS. Proteomics. 2007; 7: 2132–2141. 17549798 10.1002/pmic.200700111

[44] Yoshida M, Higashi K, Kobayashi E, Saeki N, Wakui K, Kusaka T, et al Correlation between images of silent brain infarction, carotid atherosclerosis and white matter hyperintensity, and plasma levels of acrolein, IL-6 and CRP. Atherosclerosis. 2010; 211: 475–479. 10.1016/j.atherosclerosis.2010.03.031 20417516

[45] Ishii T, Yamada T, Mori T, Kumazawa S, Uchida K, Nakayama T. Characterization of acrolein-induced protein cross-links. Free Radic Res. 2007; 41: 1253–1260. 17922343 10.1080/10715760701678652

[46] Chadwick AC, Sahoo D. Functional characterization of newly-discovered mutations in human SR-BI. PLoS One. 2012; 7: e45660 10.1371/journal.pone.0045660 23029167 PMC3448639

[47] Zhang X, Goncalves R, Mosser DM. The isolation and characterization of murine macrophages Curr Protoc Immunol. 2008; Chapter 14: Unit 14 11. 10.1002/0471142735.im0815s83 PMC2834554 19016445

[48] Lowry OH, Rosebrough NJ, Farr AL, Randall RJ. Protein measurement with the Folin phenol reagent. J Biol Chem. 1951; 193: 265–275. 14907713

[49] Connelly MA, Klein SM, Azhar S, Abumrad NA, Williams DL. Comparison of class B scavenger receptors, CD36 and scavenger receptor BI (SR-BI), shows that both receptors mediate high density lipoprotein-cholesteryl ester selective uptake but SR-BI exhibits a unique enhancement of cholesteryl ester uptake. J Biol Chem. 1999; 274: 41–47. 9867808 10.1074/jbc.274.1.41

[50] Parathath S, Connelly MA, Rieger RA, Klein SM, Abumrad NA, De La Llera-Moya M, et al Changes in plasma membrane properties and phosphatidylcholine subspecies of insect Sf9 cells due to expression of scavenger receptor class B, type I, and CD36. J Biol Chem. 2004; 279: 41310–41318. 15280390 10.1074/jbc.M404952200

[51] Parathath S, Darlington YF, de la Llera Moya M, Drazul-Schrader D, Williams DL, Phillips MC, et al Effects of amino acid substitutions at glycine 420 on SR-BI cholesterol transport function. J Lipid Res. 2007; 48: 1386–1395. 17372332 10.1194/jlr.M700086-JLR200

[52] Macala LJ, Yu RK, Ando S. Analysis of brain lipids by high performance thin-layer chromatography and densitometry. J Lipid Res. 1983; 24: 1243–1250. 6631248

[53] Mizel SB, Graff AH, Sriranganathan N, Ervin S, Lees CJ, Lively MO, et al Flagellin-F1-V fusion protein is an effective plague vaccine in mice and two species of nonhuman primates. Clin Vaccine Immunol. 2009; 16: 21–28. 10.1128/CVI.00333-08 18987167 PMC2620661

[54] Sorci-Thomas MG, Parks JS, Kearns MW, Pate GN, Zhang C, Thomas MJ. High level secretion of wild-type and mutant forms of human proapoA-I using baculovirus-mediated Sf-9 cell expression. J Lipid Res. 1996; 37: 673–683. 8728328

[55] Li HH, Thomas MJ, Pan W, Alexander E, Samuel M, Sorci-Thomas MG. Preparation and incorporation of probe-labeled apoA-I for fluorescence resonance energy transfer studies of rHDL. J Lipid Res. 2001; 42: 2084–2091. 11734582

[56] Shao B. Site-specific oxidation of apolipoprotein A-I impairs cholesterol export by ABCA1, a key cardioprotective function of HDL. Biochim Biophys Acta. 2012; 1821: 490–501. 10.1016/j.bbalip.2011.11.011 22178192 PMC3288272

[57] Xu S, Huang Y, Xie Y, Lan T, Le K, Chen J, et al Evaluation of foam cell formation in cultured macrophages: an improved method with Oil Red O staining and DiI-oxLDL uptake. Cytotechnology. 2010; 62: 473–481. 10.1007/s10616-010-9290-0 21076992 PMC2993859

[58] Shao B, Heinecke JW. HDL, lipid peroxidation, and atherosclerosis. J Lipid Res. 2009; 50: 599–601. 10.1194/jlr.E900001-JLR200 19141435 PMC2656652

[59] Gillard BK, Chen YS, Gaubatz JW, Massey JB, Pownall HJ. Plasma factors required for human apolipoprotein A-II dimerization. Biochemistry. 2005; 44: 471–479. 15641771 10.1021/bi048591j

[60] de Beer MC, Durbin DM, Cai L, Jonas A, de Beer FC, van der Westhuyzen DR. Apolipoprotein A-I conformation markedly influences HDL interaction with scavenger receptor BI. J Lipid Res. 2001; 42: 309–313. 11181762

[61] Liadaki KN, Liu T, Xu S, Ishida BY, Duchateaux PN, Krieger JP, et al Binding of high density lipoprotein (HDL) and discoidal reconstituted HDL to the HDL receptor scavenger receptor class B type I. Effect of lipid association and APOA-I mutations on receptor binding. J Biol Chem. 2000; 275: 21262–21271. 10801839 10.1074/jbc.M002310200

[62] Nieland TJ, Xu S, Penman M, Krieger M. Negatively cooperative binding of high-density lipoprotein to the HDL receptor SR-BI. Biochemistry. 2011; 50: 1818–1830. 10.1021/bi101657j 21254782 PMC3065119

[63] Gillotte-Taylor K, Boullier A, Witztum JL, Steinberg D, Quehenberger O. Scavenger receptor class B type I as a receptor for oxidized low density lipoprotein. J Lipid Res. 2001; 42: 1474–1482. 11518768

[64] Gugliucci A, Lunceford N, Kinugasa E, Ogata H, Schulze J, Kimura S. Acrolein inactivates paraoxonase 1: changes in free acrolein levels after hemodialysis correlate with increases in paraoxonase 1 activity in chronic renal failure patients. Clin Chim Acta. 2007; 384: 105–112. 17632094 10.1016/j.cca.2007.06.012

[65] Tamamizu-Kato S, Wong JY, Jairam V, Uchida K, Raussens V, Kato H, et al Modification by acrolein, a component of tobacco smoke and age-related oxidative stress, mediates functional impairment of human apolipoprotein E. Biochemistry. 2007; 46: 8392–8400. 17580963 10.1021/bi700289k PMC2556514

[66] Sakata K, Kashiwagi K, Sharmin S, Ueda S, Irie Y, Murotani N, et al Increase in putrescine, amine oxidase, and acrolein in plasma of renal failure patients. Biochem Biophys Res Commun. 2003; 305: 143–149. 12732208 10.1016/s0006-291x(03)00716-2

